# Severity of depressive but not anxiety symptoms impacts glucose metabolism among patients with type 2 diabetes in primary care

**DOI:** 10.3389/fmed.2022.944047

**Published:** 2022-07-28

**Authors:** Csenge Hargittay, Ajándék Eöry, Bernadett Márkus, András Mohos, Tamás Ferenci, Krisztián Vörös, Zoltán Rihmer, Xenia Gonda, Péter Torzsa

**Affiliations:** ^1^Department of Family Medicine, Faculty of Medicine, Semmelweis University, Budapest, Hungary; ^2^Department of Family Medicine, Faculty of Medicine, University of Szeged, Szeged, Hungary; ^3^Physiological Controls Research Center, Óbuda University John von Neumann Faculty of Informatics, Budapest, Hungary; ^4^Department of Statistics, Corvinus University of Budapest, Budapest, Hungary; ^5^Department of Psychiatry and Psychotherapy, Faculty of Medicine, Semmelweis University, Budapest, Hungary; ^6^International Centre for Education and Research in Neuropsychiatry, Samara State Medical University, Samara, Russia

**Keywords:** type 2 diabetes, depression, anxiety, primary care, screening, mental health

## Abstract

**Background:**

Data from primary care regarding the prevalence of symptoms of depression and anxiety, and their effect on glycemic control among people with diabetes is lacking in Hungary. The recently introduced Patient Health Record (PHR) requires family doctors to screen for depressive symptoms.

**Objectives:**

We aimed to investigate the prevalence of depressive and anxiety symptoms among patients with type 2 diabetes in the general practice, and the relationship between these affective disorders and glycated hemoglobin (HbA1c) level.

**Methods:**

We included 338 consecutive patients with type 2 diabetes from six primary care practices in this cross-sectional study. A self-administered questionnaire (patient history, anthropometric, socioeconomic, laboratory parameters), the Beck Depression Inventory (BDI) and the Hamilton Anxiety Scale (HAM-A) were used.

**Results:**

The mean age of the sample was 64.0 ± 11.5 (years ± SD), 61% of participants were female. The prevalence of depressive symptoms was 21%, mainly moderate/severe symptoms (13%). Anxiety symptoms were more common (35%). We found significant univariate association between the depressive symptoms and HbA1c (*p* = 0.001), suicide attempt (*p* < 0.001), anxiety (*p* < 0.001), micro- and macrovascular complication (*p* = 0.028 and *p* < 0.001), education (*p* = 0.001) and place of residence (*p* = 0.002). In multivariate analysis, however, only BDI score had significant (*p* = 0.03191) association with glycemic control.

**Conclusion:**

Among primary care patients with type 2 diabetes, the prevalence of depressive symptoms was less frequent than anxiety symptoms. More severe depressive symptoms were associated with worse glycemic control.

## Introduction

Diabetes, depression and anxiety are major public health issues due to their high prevalence and dire consequences. Untreated long-term depression can worsen not only the mental status and increase the suicide risk of the patient but is also associated with a number of chronic conditions. Comorbidity with other physical diseases results in worse state of health than depression alone, or any specific chronic condition by itself, or in combination with other chronic diseases excluding depression. The comorbidity between depression and diabetes has been proven to be the most detrimental combination (1).

The prevalence of depression among patients with type 2 diabetes is two times higher than in persons without diabetes (2) and is often undiagnosed and untreated (3).

Anxiety disorders are also more common in patients with diabetes. According to a meta-analysis, anxiety symptoms, and disorders are by 25% more common among patients with diabetes than in control populations (4).

The co-occurrence of depression or anxiety with diabetes can increase morbidity. Diabetic patients with anxiety and/or depression have lower adherence to treatment, worse quality of life, and the number of patients living with disabilities is also increased (4, 5).

Type 2 diabetes mellitus is commonly diagnosed, treated and managed in general practice (6). Screening for depressive and anxiety symptoms and timely treatment among diabetic patients could lead to a more efficient control of glucose metabolism, provided that depression and anxiety have a direct detrimental effect on diabetes control in this setting, and if screening is active enough to find the majority of these patients.

However, studies evaluating such a direct deleterious effect of depression and anxiety on diabetes yielded contradictory results. Some studies found significant correlation between depression and HbA1c levels in patients with type 2 diabetes (7, 8), while others reported no association between these two factors (9, 10).

Evidence suggests that, like depression, anxiety may have controversial associations with glycemic control. Anderson et al. reported a significant association between anxiety symptoms and higher HbA1c (11), while another study did not detect such a relationship (12).

Primary care is the ideal setting for screening, however, several barriers do exist. Limited training, and available time for screening could be doctor related, while access to specialist care and screening tools are practice level difficulties (13).

The Patient Health Record has been introduced in Hungary in primary care, in hope that it can help to eliminate the above barriers. The Health Record requires extended medical data collection about patients, including the shortened Beck Depression Inventory. Thus, this measurement provides an excellent opportunity to systematically screen for depressive symptoms among people with diabetes in primary care.

The aims of our study were:

to assess the prevalence of depressive and anxiety symptoms using standardized questionnaire among patients with type 2 diabetes in general practice settings, and

to investigate whether the presence of depressive and anxiety symptoms is related to higher HbA1c level and thus directly deteriorates diabetes control.

## Materials and methods

### Patients

In a cross-sectional design, 338 consecutive Caucasian adult patients with type 2 diabetes mellitus were enrolled from six primary care practices in Hungary between September 2018 and February 2020. Physicians of the practices voluntarily took part in the study and helped enrolling their patients in the study. Exclusion criteria were type 1 diabetes mellitus, gestational diabetes, patients with severe cognitive disorder and antidepressant treatment. Only family physicians had access to patient-level data. Clinical data sheets and filled out questionnaires were marked by code numbers and further analyzed by the investigators.

Patients received a thorough oral and written explanation of the study. Prior to enrollment, all participating patients gave written informed consent. The study was approved by the national ethical committee (44677-2/2018/EKU) and was carried out in accordance with the tenets of the Declaration of Helsinki.

### Measures

A self-administered questionnaire was used to assess sociodemographic characteristics (age, gender and education), physical exercise, addictive behaviors, history of attempted suicide. Trained personnel of the practices were involved to undertake physical examination and to record anthropometric parameters.

General practitioners (GPs) provided data on the presence of chronic and psychiatric diseases, micro- and macrovascular complications. Existing laboratory parameters were obtained from the medical records of patients (HbA1c, blood sugar, serum lipids).

#### Beck Depression Inventory

The Beck Depression Inventory is a 21-question multiple-choice self-report questionnaire, widely used for measuring the severity of depressive symptoms. Participants make ratings on a four-point Likert scale (0–3), where a higher score indicates more severe symptoms (14). Cut-off points for rating the severity of depressive symptoms are as follows:

0–13: no, or minimal,

14–18: mild,

19–24: moderate,

above 25 points: severe symptoms.

#### Hamilton Anxiety Scale

The Hamilton Anxiety Scale (HAM-A) was used to assess the severity of anxiety. The scale consists of 14 items, each of which is scored on a scale of 0 (not present) to 4 (severe anxiety) (15). Cut-off points for rating the severity of anxiety are as follows:

0–13: no, or minimal,

14–17: mild,

18–24: moderate,

25 points or above: severe symptoms.

### Statistical analysis

Categorical data are presented as count (percentage) and are compared univariately among groups with Chi-square test, continuous data are presented as mean ± standard deviation, and are compared univariately with Mann-Whitney *U*-test.

Multivariable analysis was carried out to control for potential confounding factors. Response variable was HbA1c, with age, gender, HAM-A score, BDI score, Body Mass Index (BMI), alcohol consumption, physical exercise, smoking, and level of education being the predictor variables. A linear regression model was used (16). The potential non-linearity in the continuous covariates of the model was checked with restricted cubic spline expansion, but was deemed non-significant (*p* = 0.4747), as was the interaction between BDI score and alcohol consumption, physical exercise and smoking (*p* = 0.4431; joint test for non-linearity and interaction: *p* = 0.4639). As the resulting model is therefore purely linear, the results are presented as estimated coefficients with 90, 95, and 99% percent confidence intervals.

A statistical power analysis was performed for sample size estimation using G*Power (17). With medium effect size (0.15), with an alpha of 0.05 and power of 0.95, and calculating with the 10 predictors in linear regression analysis, the projected minimum sample size needed was 172.

All data were analyzed using R statistical environment version 4.1.0 using package rms version 6.2-0 (The R Project for Statistical Computing).

## Results

### Characteristics of patients

A total of 344 patients were approached by their physicians. One person was excluded due to having type 1 diabetes, and five persons declined participation (three for not having time, two adults not providing specific explanation). A total of 338 patients with type 2 diabetes participated in our study, their descriptive data is presented in Table 1.

**TABLE 1:** Basic description of the sample in primary care, Hungary (2018–2020, *n* = 338).

	Total *n* = 338
Age (years)	64.0 ± 11.5
Female	207 (61%)
**Education**	
Elementary	110 (33%)
Secondary	125 (37%)
Tertiary	101 (30%)
**Residence**	
Capital city	173 (51%)
City	142 (42%)
Village	23 (7%)
Alcohol consumption	120 (36%)
Smokers	45 (13%)
Regular physical exercise	71 (21%)
Suicide attempt	10 (3%)
Glycated hemoglobin (%)	7.23 ± 1.26
Blood glucose (mmol/l)	8.39 ± 4.11
seTG (mmol/l)	2.068 ± 1.295
seChol (mmol/l)	4.89 ± 1.26
LDL (mmol/l)	2.62 ± 1.06
HDL (mmol/l)	1.38 ± 0.63
BMI	31.09 ± 5.91
Normal BMI	45 (13%)
Overweight (25–29.9)	112 (33%)
Obese (≥30)	180 (53%)
Microvascular complications	92 (27%)
Macrovascular complications	131 (39%)
**Beck Depression Inventory**	
No, or minimal symptoms	265 (79%)
Mild	26 (8%)
Moderate	31 (9%)
Severe symptoms	14 (4%)
BDI	8.49 ± 7.65
**Hamilton Anxiety Scale**	
No, or minimal symptoms	221 (65%)
Mild	50 (15%)
Moderate	38 (11%)
Severe symptoms	29 (9%)
HAM-A	11.08 ± 8.65

The mean age of the sample was 64.0 ± 11.5 years and consisted of more females (61%). The level of education was evenly dispersed, 33% of the patients graduated from elementary school, 30% had a university degree and 37% graduated from high school.

The mean BMI was 31.09 ± 5.91 kg/m^2^ and the majority of participants were obese (53%). The consumption of alcohol was common (36% drinking regularly), but the proportion of smokers was relatively low (13%).

The mean HbA1c was 7.23 ± 1.26%. A total of 27% of the patients had microvascular and 39% had macrovascular complications.

Depressive symptoms, as measured by BDI were present in 21% of patients, 13% of the whole sample had moderate/severe symptoms. We found anxiety symptoms in one third (35%) of the patients, 15% had mild, 11% had moderate, and 9% had severe symptoms.

### The comparison of patients with type 2 diabetes by depressive symptoms severity

For data presentation purposes, we divided the patients into two groups: no, or minimal/mild and moderate/severe depressive symptoms groups, according to their depressive symptoms (Table 2). Patients with moderate/severe depressive symptoms had significantly poorer glucose control. Depressive symptoms were more common among less educated patients. They had higher anxiety score and had more suicide attempts and micro- and macrovascular complications. Patients living in the capital city had lower depressive symptoms scores.

**TABLE 2:** The comparison of demographic and clinical characteristics of patients with type 2 diabetes by depressive symptoms severity in Hungary (*n* = 338, 2018–2020).

Variable	Depressive symptoms		*P*-value
	No, minimal and mild *n* = 291	Moderate and severe *n* = 47	
Age (years)	63.9 ± 11.7	64.3 ± 10.1	0.795**
Female	173 (59%)	34 (72%)	0.092*
Education			0.001*
Elementary	**84 (29%)**	**26 (55%)**	
Secondary	111 (38%)	14 (30%)	
Tertiary	**94 (33%)**	**7 (15%)**	
Residence			0.002*
Capital city	**159 (55%)**	**14 (30%)**	
City	**116 (40%)**	**26 (55%)**	
Village	**16 (5%)**	**7 (15%)**	
Alcohol consumption	102 (35%)	18 (38%)	0.666*
Smokers	36 (12%)	9 (19%)	0.204*
Regular physical exercise	66 (23%)	5 (11%)	0.057*
Suicide attempt	5 (2%)	5 (11%)	**<0.001**
Glycated hemoglobin (%)	7.16 ± 1.27	7.65 ± 1.14	0.001**
Blood sugar (mmol/l)	8.32 ± 4.26	8.79 ± 3.00	0.26**
seTG (mmol/l)	2.07 ± 1.34	2.05 ± 0.98	0.5**
seChol (mmol/l)	4.87 ± 1.27	5.00 ± 1.17	0.67**
LDL (mmol/l)	2.59 ± 1.09	2.82 ± 0.89	0.157**
HDL (mmol/l)	1.38 ± 0.65	1.36 ± 0.45	0.988**
BMI	31.17 ± 6.04	30.56 ± 5.00	0.715**
Normal BMI	40 (14%)	5 (11%)	0.876*
Overweight (25–29.9)	94 (32%)	18 (38%)	
Obese (≥30)	156 (54%)	24 (51%)	
Microvascular complications	73 (25%)	19 (40%)	0.028*
Macrovascular complications	101 (35%)	30 (64%)	<0.001*
Hamilton Anxiety Scale			<0.001*
No, or minimal Symptoms	**213 (73%)**	**8 (17%)**	
Mild	39 (13%)	11 (23%)	
Moderate	**26 (9%)**	**12 (26%)**	
Severe symptoms	**13 (4%)**	**16 (34%)**	
HAM-A	9.49 ± 7.20	20.94 ± 10.26	<0.001**

Significant p-values and significant difference based on adjusted standardized residuals are marked in bold. *Chi-square test. **Mann–Whitney U-test.

Regular physical exercise was lower in patients with moderate/severe depressive symptoms and there were more women among them, however, these factors were not significant (*p* = 0.057 and 0.092, respectively).

### Multivariable modeling of hemoglobin

**FIGURE 1:**
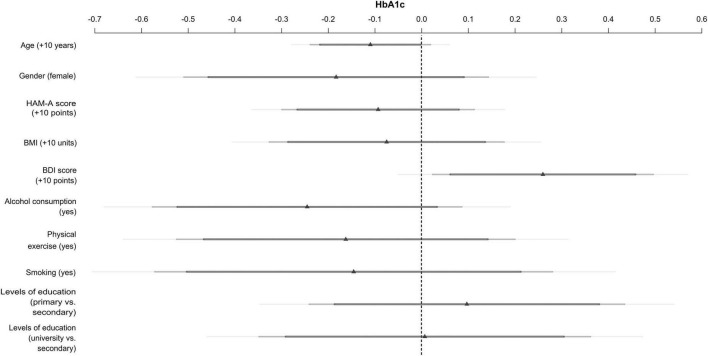
Impact of depressive (BDI score) and anxiety (HAM-A score) symptoms on HbA1c, controlling for age, gender, Body Mass Index (BMI), alcohol consumption, physical exercise, smoking, and levels of education in patients with type 2 diabetes using multivariable linear regression (Hungary, *n* = 338, 2018–2020).

Results of the multivariable model are shown on Figure 1, and are numerically given in Table 3. Only the effect of the BDI score was significant (*p* = 0.03191), but it was significant even after adjusting for a number of control variables (including HAM-A score, age, gender, BMI, alcohol consumption, physical exercise, smoking, and levels of education). A 10-unit increase in BDI was associated with a 0.26% point increase in HbA1c (95% confidence interval: 0.02–0.50).

**TABLE 3:** Results of multivariable modeling of HbA1c (Hungarian patients with type 2 diabetes, 2018–2020, *n* = 338).

Variable	Effect	95% confidence interval	*p*-value
Age (+ 10 years)	−0.11	−0.24–0.02	0.09492
Gender (female)	−0.18	−0.51–0.14	0.27164
HAM−A score (+ 10 points)	−0.09	−0.3–0.11	0.37567
BMI (+ 10 units)	−0.08	−0.33–0.18	0.55900
BDI score (+ 10 points)	0.26	0.02–0.50	**0.03191**
Alcohol consumption (yes)	−0.25	−0.58–0.09	0.14776
Physical exercise (yes)	−0.16	−0.53–0.20	0.37994
Smoking (yes)	−0.15	−0.57–0.28	0.50353
Levels of education (primary vs. secondary)	0.10	−0.24–0.44	0.83175
Levels of education (tertiary vs. secondary)	0.01	−0.35–0.36	0.83175

Significant p value is marked in bold.

## Discussion

To our knowledge, this was the first study in Hungary which estimated the prevalence of depressive and anxiety symptoms in a primary care sample of people with type 2 diabetes. The occurrence of depressive symptoms measured by the BDI was 21% (mild/moderate/severe combined) and the prevalence of anxiety measured by the HAM-A was 35%. We found a positive correlation between the BDI score and HbA1c, more severe depressive symptoms were associated with worse glucose control.

The high prevalence of depression among patients with type 2 diabetes has been reported in several studies. According to a systematic review and meta-analysis of 248 observational studies (*n* = 83 020 812), the prevalence of comorbid depression in patients with type 2 diabetes was 28% in the world, 27% in Africa, 28% in America, 29% in Australia, 32% in Asia, and the lowest in Europe, with 24% (18). In India the prevalence was higher, between 27 and 49% (19). European studies have reported wide variations in the prevalence of depression in patients with type 2 diabetes (20–23). In Italy and Greece, the prevalence was lower, 18.6 and 33.4% (20, 21), while in Central Eastern Europe (Poland, Slovakia) the prevalence was somewhat higher (29.7 and 53%) (22, 23). According to the data from Eurostat, the prevalence of depression in Hungary was 4.0% in 2019 (24). Hungary was in the lowest tertile on this list of European countries, ranking 24th, which aligns well with our results. A study conducted in Hungarian primary care found that the current prevalence of depressive symptoms measured by the BDI and PRIME-MD (Primary Care Evaluation of Mental Disorders) was 18.5% and the prevalence of major depressive episode was 7.3% (25). The slightly higher occurrence of depressive symptoms among people with diabetes found in our study (21%) is in line with these results of relatively low prevalence, taking into consideration that these symptoms are more common among patients with diabetes (2).

Anxiety symptoms were more frequent than depression in our sample. An international study reported a prevalence of 18% of anxiety disorder among people with type 2 diabetes in 15 different countries (*n* = 3170) using the Mini-International Neuropsychiatric Interview. The prevalence was the highest in Ukraine, Saudi Arabia and Argentina (72.7, 52.2, and 37.6%, respectively), in Bangladesh and India the lowest (0.5 and 0.0%, respectively). In European countries, the prevalence of anxiety was 12.7% in Germany, 8.9% in Italy, 16.6% in Poland and 2.5% in Serbia (26). In India the prevalence rate of anxiety was 27.6%, using Hamilton Anxiety Rating Scale and it was twice as high compared to a healthy control group (27). Several European studies have also reported a prevalence rate of anxiety in diabetic patients. In Europe the highest prevalence was observed in Croatia (51.5%) (28), while lower rates were reported from Italy (14.5%) (20) and Germany (25.2%) (29). According to a Hungarian study, conducted in primary care, the lifetime and point prevalence rates of DSM-IIIR (Diagnostic and Statistical Manual of Mental Disorders) anxiety disorders were 18 and 8%, respectively (30). The prevalence of 35% among diabetic patients in our study is in the higher range of the European data. Similarly to depression, anxiety symptoms were more frequent among diabetic patients in our sample than in the Hungarian general population mentioned above, which is in line with previous studies reporting higher prevalence of depression and anxiety among people with diabetes (2, 4).

We also investigated the effect of depressive and anxiety symptoms on glycemic control. The severity of depressive symptoms measured by the BDI were significantly associated with glycemic control (*p* = 0.03191). Diabetic patients with moderate/severe symptoms of depression had significantly worse glycemic control. In multivariable analysis, a 10-units increase in BDI score was associated with a 0.26% points increase in HbA1c. The degree of anxiety symptoms, however, was not related to HbA1c concentrations (*p* = 0.37567).

Our finding is in line with results from a number of studies (7, 8), although contradictory results have also been reported (9, 10). A meta-analysis investigating 24 studies, found that in diabetic patients depression was significantly associated with glycemic control (7). Similarly, in their longitudinal study, Richardson et al. reported that the presence of depression was positively associated with HbA1c levels over time among veterans with type 2 diabetes (8).

In contrast, Fisher et al. did not find a relationship between glucose metabolism and clinical depression or depressive symptoms (9). Ahmadieh et al. also did not find a significant association between depression and glycemic control (10).

The severity of anxiety symptoms was not significantly associated with HbA1c in our sample. Anxiety is often a temporary situation compared to depression, which explains why there was no association between anxiety and poor glycemic control. Whitworth et al. similarly failed to find an association between anxiety and glycemic control but comorbid depression and anxiety were significantly associated with higher HbA1c levels (12). In contrast, Anderson et al. found a positive correlation between anxiety and glycemic control (11).

In our study, several sociodemographic characteristics were related to the presence of depressive symptoms. Higher education (33% vs. 15%, *p* = 0.001) and living in the capital city (55% vs. 30%, *p* = 0.002) seemed to be protective factors against depressive symptoms. In a recent epidemiological analysis, diabetes and depression were associated with lower educational level (31). Probst et al. reported slightly higher prevalence of depression among rural than urban areas. Poorer health status, the more frequent occurrence of chronic diseases and poverty in rural areas have been suggested as possible explanations (32). In Hungary there are several regions—especially in the countryside—where there are unfilled general practices, which may also contribute to the limited access to primary care (33). This might be also a possible explanation of higher prevalence of depressive symptoms.

In Hungary 1,706 people died due to suicide in 2020 (34). Nock et al. assessed the data of 17 countries, and found a 2.7% lifetime prevalence of suicide attempts in the general population (35). In our study, people with type 2 diabetes had a slightly higher prevalence of suicide attempts (3%) than this results. In line with our findings, a 45% higher prevalence of suicide attempt was reported among diabetic patients, and they were more often suicidal than non-diabetic patients (36). As expected, we found a striking difference between the prevalence of previous suicide attempts between the no/minimal/mild and moderate/severe depressive symptoms groups (2% vs. 11%, < 0.001). There could be hidden a truly high risk of suicide among people with diabetes and depression behind the slightly elevated suicide risk among all people with diabetes. Based on our findings, screening for depressive symptoms and adequate treatment (antidepressant medication use and psychotherapy) could be crucial among people with diabetes to prevent suicide attempts.

The association between depression and diabetes complications is inconsistent. Some studies found that the presence of depression is associated with micro- and macrovascular complications (37, 38). Our findings are similar to these studies, as micro- and macrovascular complications were significantly higher in patients with moderate/severe depressive symptoms in our cohort (25% vs. 40% *p* = 0.028 and 35% vs. 64%, *p* < 0.001).

Depression is associated with certain behaviors which may carry its impact on glycemic control. Depression in patients with type 2 diabetes might have a negative impact on self-care behaviors, which could at least in part explain its deleterious effect on glycemic control. Depression has been reported to have a negative effect on regular physical activity, medication adherence and diet (5). In our study regular physical exercise was lower in patients with moderate/severe depressive symptoms (11% vs. 23%), although this association was not statistically significant (*p* = 0.057).

Depressive symptoms have been reported to be more common among women (21). In our sample there were more women in the moderate/severe depressive symptoms group (59% vs. 72%), however, the difference was not significant (*p* = 0.092). We also found no significant difference in age (63.9 ± 11.7 vs. 64.3 ± 10.1, *p* = 0.795). Tran et al. found that the prevalence of depression in type 2 diabetic patients was significantly higher in patients < 60 years old than among patients ≥ 60 years old. Suggested explanations were the more common occurrence of marital and work difficulties among active patients (39).

A recent study reported a significant association between the BMI and depression among type 2 diabetic patients (40). In our study, we found no significant differences in BMI among patients with moderate/severe depressive symptoms (31.17 ± 6.04 vs. 30.56 ± 5.00, *p* = 0.715).

The usefulness of depression screening in the general adult population is controversial and differs between countries (41), but its importance is unquestionable in patients with type 2 diabetes (42–44). The International Diabetes Federation and the American Diabetes Association (ADA) emphasize the psychological aspects of diabetes and the screening for mental disorders in this population (42, 43). The ADA recommends annual screening of all diabetic patients, especially of those who disclose depression in their self-reported history (43). The Hungarian Clinical Practice Guideline also recommends assessing mental status, such as depression (44).

The new PHR has recently been introduced in primary care. It requires family doctors to collect comprehensive health data about their patients, including complete history, mental, and physical state, lifestyle, etc. The PHR created a great opportunity to screen for depressive symptoms among patients with type 2 diabetes mellitus. The high prevalence of depressive symptoms and the marked increase in suicide attempts in our sample underlines the importance of screening for depressive symptoms among people with diabetes in primary care. The PHR includes the short versions of the Beck Hopelessness Scale and the Beck Depression Inventory. An additional recommendation is also available that helps general practitioners to screen and recognize not only depressive symptoms but acute suicide risk as well (45).

The direct association between depressive symptoms and glycemic control in our study raises the possibility that screening might even lead to improved outcomes in diabetes, as effective depression treatment may result in better control of diabetes. Brieler et al. found that antidepressant use was associated with improved glycemic control among patients with depression and type 2 diabetes (46). Collaborative care also improves outcomes for depression. In the TEAMcare study, Katon et al. found the collaborative care in the intervention group caused improvement in HbA1c levels, depression scores, LDL cholesterol levels and systolic blood pressure over a 12-month treatment period (47). This model was implemented in India by Ali et al. The results of the INDEPENDENT (Integrating Depression and Diabetes Treatment) study also found that collaborative care improved depression and cardiometabolic indices among patients with diabetes and depression at 24 months (48). A secondary analysis of the INDEPENDENT trial by Kemp et al. showed that collaborative intervention for the treatment of depression and diabetes can lead to reduction in anxiety symptoms among patients with anxiety (49).

This study has methodological limitations such as lack of control group and relatively small sample size, our cohort might not be epidemiologically valid. Another limitation is the cross-sectional design, and thus, the examination of a causal relationship between clinical variables and depressive or anxiety symptoms cannot be determined. Symptoms of depression and anxiety have been detected by BDI and HAM-A screening method and these diagnoses were not based on standard clinical diagnostic criteria like DSM-5 or ICD-10 (International Statistical Classification of Diseases-10).

The comparability of our study with the others discussed above could be limited. There is a wide variation between the prevalence rate of comorbid depression and anxiety in diabetic patients reported in different studies, which could be due to differences in study design, patient-, disease-, and healthcare-related factors, or diagnostic tools used.

## Conclusion

In our sample of Hungarian primary care patients with type 2 diabetes, the prevalence of depressive symptoms was less frequent than anxiety symptoms, however, a relatively large number of patients in the former group had suicide attempt. More severe depressive symptoms were associated with worse glycemic control. Whether the screening introduced in Hungary could lead to better outcomes in depression and diabetes treatment warrants further studies.

## Data availability statement

The raw data supporting the conclusions of this article will be made available by the authors, without undue reservation.

## Ethics statement

The studies involving human participants were reviewed and approved by the Semmelweis University Ethics Committee. The patients/participants provided their written informed consent to participate in this study.

## Author contributions

CH: writing the manuscript. PT and XG: study design. AE, CH, BM, AM, PT, and KV: data collection. TF: provide statistical analysis. AE, XG, BM, AM, TF, KV, ZR, and PT: review and edit the manuscript. XG, PT, and ZR: critical revision of the article. All authors contributed to the article and approved the submitted version.

## Conflict of interest

The authors declare that the research was conducted in the absence of any commercial or financial relationships that could be construed as a potential conflict of interest.

## Publisher’s note

All claims expressed in this article are solely those of the authors and do not necessarily represent those of their affiliated organizations, or those of the publisher, the editors and the reviewers. Any product that may be evaluated in this article, or claim that may be made by its manufacturer, is not guaranteed or endorsed by the publisher.

## Abbreviations

ADA: American Diabetes Association
BDI: Beck Depression Inventory
BMI: Body Mass Index
DSM-IIIR: Diagnostic and Statistical Manual of Mental Disorders
HAM-A: Hamilton Anxiety Scale
ICD-10: International Statistical Classification of Diseases-10
PHR: Patient Health Record
PRIME-MD: Primary Care Evaluation of Mental Disorders.
